# Supplementary material for: The Associations between Liver Enzymes and Cardiovascular Risk Factors in Adults with Mild Dyslipidemia
Source: J Clin Med. 2020 Apr 17;9(4):1147. doi: 10.3390/jcm9041147 (PMC7230762; doi:10.3390/jcm9041147)
Supplement: Supplementary file 1 [file jcm-09-01147-s001.pdf]

The associations between liver enzymes and cardiovascular risk factors in adults with mild dyslipidemia

Eun-Ock Park[1,†], Eun-Ju Bae[2,†], Byung-Hyun Park[3,*] and Soo-Wan Chae[1,*]

[1]Clinical Trial Center for Functional Foods, Jeonbuk National University Hospital, Jeonju, Jeonbuk 54907, Republic of Korea

[2]College of Pharmacy, Jeonbuk National University Hospital, Jeonju, Jeonbuk 54896, Republic of Korea

[3]Department of Biochemistry, Jeonbuk National University Medical School, Jeonju, Jeonbuk 54896, Republic of Korea

Contents

1. Supplementary Figure
2. Supplementary Tables

Figure S1

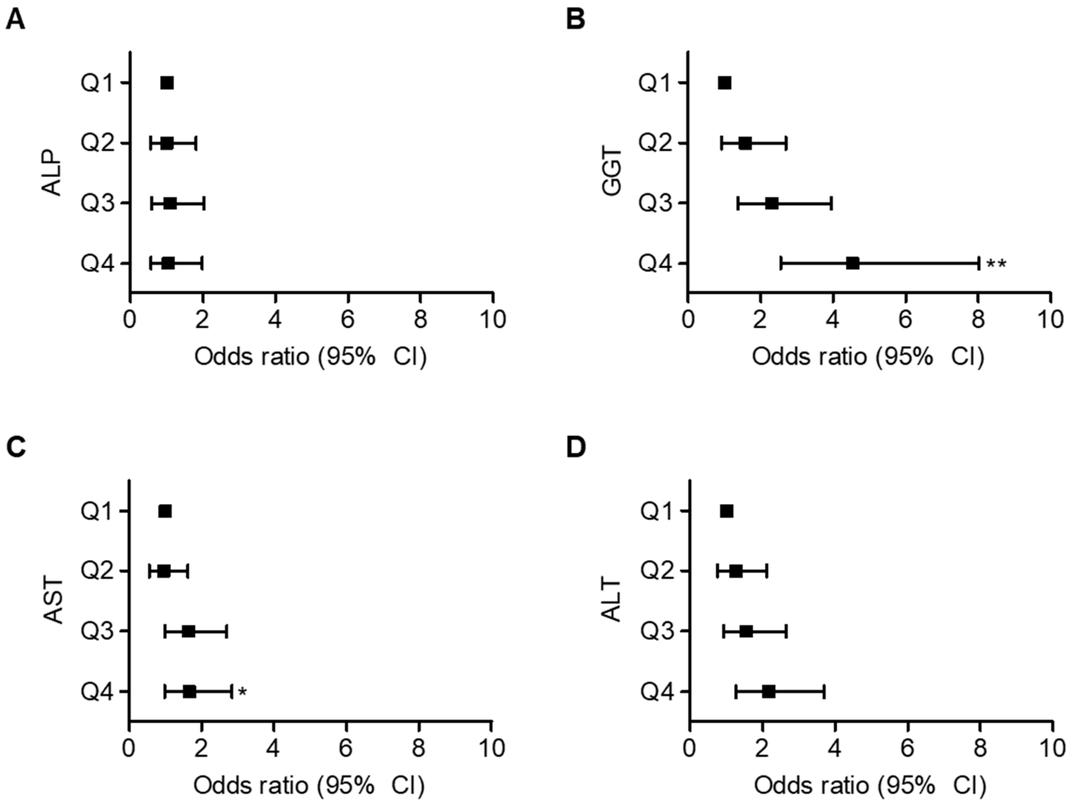

Figure S1. ORs with 95% CI for hypertension stratified by unadjusted liver enzymes quartile. The quartile with the lowest liver enzymes is used as reference. Q1 indicates first quartile and so on. (A) ALP, p=0.994; (B) GGT, p<0.001; (C) AST, p=0.035; (D) ALT, p=0.067. $^{*}p<0.05$ and $^{**}p<0.01$ vs. Q1. Analyzed by Logistic regression analysis. Hosmer and Lemeshow's goodness of fit test ($x^2>0.05$). Abbreviation: ALP, alkaline phosphatase; GGT, gamma-glutamyltransferase; AST, aspartate aminotransferase; ALT, alanine aminotransferase; OR, odds ratio; Cl, confidence interval.

Table S1. Cardiovascular risk factors along the ALP quartiles

	Quartiles				*p-value* [1)]	*p for trend* [2)]	*Adj. p-value* [3)]
	First (Q1)	Second (Q2)	Third (Q3)	Fourth (Q4)			
SBP (mmHg)	122.17 ± 12.12	124.21 ± 13.37	126.08 ± 12.69	124.81 ± 12.57	0.249	0.149	0.001
DBP (mmHg)	80.18 ± 10.06	81.21 ± 10.52	81.47 ± 10.72	80.14 ± 9.18	0.763	0.863	0.0001
TC (mg/dl)	210.76 ± 26.72	213.98 ± 24.90	212.18 ± 27.70	223.20 ± 28.41	0.013	0.031	0.789
TG (mg/dl)	149.55 ± 52.77	168.33 ± 71.01	167.99 ± 60.53	167.11 ± 66.49	0.141	0.071	0.001
HDL-C (mg/dl)	51.43 ± 12.84	49.42 ± 10.50	48.92 ± 10.48	48.46 ± 9.95	0.304	0.159	0.089
LDL-C (mg/dl)	133.40 ± 26.03[b]	135.85 ± 23.47[ab]	134.42 ± 23.82[ab]	144.61 ± 26.72[a]	0.015	0.025	0.220
Non HDL-C (mg/dl)	159.34 ± 25.81	164.56 ± 23.59	163.26 ± 22.88	174.74 ± 26.46	0.001	0.001	0.355
VLDL (mg/dl)	31.63 ± 7.85[b]	35.28 ± 9.42[b]	33.67 ± 7.41[ab]	35.69 ± 9.86[a]	0.177	0.089	0.040
ApoA1 (g/l)	1.43 ± 0.26	1.45 ± 0.23	1.42 ± 0.24	1.41 ± 0.22	0.811	0.905	0.010
ApoB (g/l)	1.17 ± 0.21[b]	1.19 ± 0.17[ab]	1.18 ± 0.17[ab]	1.26 ± 0.22[a]	0.016	0.201	0.604
TC/HDL-C	4.28 ± 0.91[b]	4.47 ± 0.83[ab]	4.46 ± 0.76[ab]	4.75 ± 0.95[a]	0.006	0.006	0.043
LDL-C/HDL-C	2.72 ± 0.72[b]	2.85 ± 0.67[ab]	2.85 ± 0.71[ab]	3.08 ± 0.78[a]	0.010	0.012	0.017
TG/HDL-C	3.15 ± 1.50	3.64 ± 1.95	3.65 ± 1.68	3.70 ± 1.98	0.147	0.065	0.042
ApoB/ApoA1	0.85 ± 0.22	0.85 ± 0.19	0.85 ± 0.19	0.92 ± 0.22	0.096	0.102	0.061
hs-CRP[†] (mg/l)	1.03 ± 2.20	0.59 ± 0.87	0.99 ± 1.80	1.27 ± 1.99	0.100	0.026	0.164

Abbreviation: ALP, alkaline phosphatase; SBP, systolic blood pressure; DBP, diastolic blood pressure; TC, total cholesterol; TG, triglyceride; HDL-C, HDL cholesterol; LDL-C, LDL cholesterol; VLDL, very low density lipoprotein; ApoA1, apolipoprotein A1; ApoB, apolipoprotein B; hs-CRP, high sensitivity C-reactive protein. Values are presented as mean±SD. Means with the same letter are not significantly different in row by Bonferroni post hoc test (p>0.05). [†] Log transformed for comparisons to their natural units for presentation. [1)] Analyzed by one-way ANOVA. [2)] Analyzed by Jonckheere-Terpstra trend analysis. [3)] Analyzed by one-way ANOVA, adjusted for alcohol consumption[†]

Table S2. Cardiovascular risk factors along the GGT quartiles

	Quartiles				*p-value* [1)]	*p for trend* [2)]	*Adj. p-value* [3)]
	First (Q1)	Second (Q2)	Third (Q3)	Fourth (Q4)			
SBP (mmHg)	118.32 ± 13.67[c]	121.70 ± 13.41[bc]	124.42 ± 12.12[ab]	128.39 ± 12.25[a]	<.0001	<.0001	<.0001
DBP (mmHg)	76.52 ± 10.61[c]	79.07 ± 9.39[bc]	80.46 ± 9.26[b]	84.77 ± 10.09[a]	<.0001	<.0001	<.0001
TC (mg/dl)	216.51 ± 26.22	216.79 ± 24.57	214.84 ± 24.93	217.05 ± 25.79	0.919	0.699	0.552
TG (mg/dl)	129.38 ± 54.39[c]	151.35 ± 62.31[bc]	156.90 ± 61.70[b]	190.38 ± 69.85[a]	<.0001	<.0001	<.0001
HDL-C (mg/dl)	55.17 ± 12.50[a]	51.78 ± 13.08[ab]	49.05 ± 9.39[bc]	47.37 ± 9.84[c]	<.0001	<.0001	0.0002
LDL-C (mg/dL)	138.37 ± 25.01	138.45 ± 23.15	137.85 ± 24.18	139.22 ± 23.79	0.981	0.990	0.066
Non HDL-C (mg/dl)	161.34 ± 24.70	165.01 ± 23.78	165.78 ± 23.62	169.68 ± 24.18	0.079	0.023	0.471
VLDL (mg/dl)	33.56 ± 8.18[b]	29.47 ± 6.13[b]	33.22 ± 6.79[b]	39.21 ± 10.42[a]	<.0001	0.001	0.017
ApoA1 (g/l)	1.49 ± 0.24	1.48 ± 0.26	1.41 ± 0.21	1.42 ± 0.25	0.042	0.006	0.001
ApoB (g/l)	1.18 ± 0.21	1.21 ± 0.19	1.22 ± 0.20	1.25 ± 0.19	0.047	0.010	0.297
TC/HDL-C	4.08 ± 0.83[c]	4.37 ± 0.88[bc]	4.50 ± 0.80[ab]	4.74 ± 0.96[a]	<.0001	<.0001	0.001
LDL-C/HDL-C	2.62 ± 0.69[b]	2.80 ± 0.72[ab]	2.89 ± 0.66[ab]	3.06 ± 0.79[a]	<.0001	<.0001	0.001
TG/HDL-C	2.56 ± 1.43[c]	3.20 ± 1.76[bc]	3.39 ± 1.65[b]	4.27 ± 2.05[a]	<.0001	<.0001	0.0002
ApoB/ApoA1	0.81 ± 0.20[b]	0.85 ± 0.19[ab]	0.88 ± 0.20[ab]	0.91 ± 0.22[a]	0.003	0.0003	0.004
hs-CRP[†] (mg/l)	0.79 ± 2.06[b]	0.96 ± 2.21[ab]	0.69 ± 1.00[ab]	1.10 ± 1.79[a]	0.006	0.0003	0.091

Abbreviation: GGT, gamma-glutamyltransferase; SBP, systolic blood pressure; DBP, diastolic blood pressure; TC, total cholesterol; TG, triglyceride; HDL-C, HDL cholesterol; LDL-C, LDL cholesterol; VLDL, very low density lipoprotein; ApoA1, apolipoprotein A1; ApoB, apolipoprotein B; hs-CRP, high sensitivity C-reactive protein. Values are presented as mean±SD. Means with the same letter are not significantly different in row by Bonferroni post hoc test (p>0.05). [†] Log transformed for comparisons to their natural units for presentation
[1)] Analyzed by one-way ANOVA. [2)] Analyzed by Jonckheere-Terpstra trend analysis. [3)] Analyzed by one-way ANOVA, adjusted for alcohol consumption[†]

Table S3. Cardiovascular risk factors along the AST quartiles

	Quartiles				*p-value* [1)]	*p for trend* [2)]	*Adj. p-value* [3)]
	First (Q1)	Second (Q2)	Third (Q3)	Fourth (Q4)			
SBP (mmHg)	122.82 ± 13.72	120.83 ± 13.11	122.92 ± 12.83	125.59 ± 13.63	0.116	0.142	0.004
DBP (mmHg)	79.46 ± 10.68	78.31 ± 9.29	80.65 ± 9.92	81.91 ± 10.89	0.091	0.031	0.001
TC (mg/dl)	214.90 ± 24.66	219.65 ± 24.44	217.09 ± 24.61	214.11 ± 27.96	0.425	0.513	0.351
TG (mg/dl)	141.73 ± 52.24[b]	141.49 ± 56.01[b]	162.49 ± 64.08[ab]	181.86 ± 82.61[a]	<.0001	<.0001	0.0004
HDL-C (mg/dl)	50.32 ± 11.75	53.08 ± 10.43	51.54 ± 12.44	49.28 ± 11.58	0.134	0.459	0.521
LDL-C (mg/dl)	138.93 ± 21.54	140.33 ± 24.17	138.46 ± 24.88	136.04 ± 26.21	0.671	0.153	0.158
Non HDL-C (mg/dl)	164.58 ± 22.16	166.57 ± 23.03	165.55 ± 25.21	164.82 ± 26.97	0.937	0.715	0.435
VLDL (mg/dl)	33.23 ± 6.21	34.00 ± 11.57	32.57 ± 8.99	36.01 ± 8.91	0.377	0.137	0.225
ApoA1 (g/l)	1.42 ± 0.22	1.47 ± 0.22	1.47 ± 0.26	1.45 ± 0.26	0.356	0.498	0.036
ApoB (g/l)	1.20 ± 0.20	1.22 ± 0.19	1.20 ± 0.19	1.24 ± 0.22	0.505	0.526	0.722
TC/HDL-C	4.43 ± 0.84	4.27 ± 0.83	4.41 ± 0.97	4.51 ± 0.94	0.314	0.537	0.682
LDL-C/HDL-C	2.89 ± 0.71	2.73 ± 0.66	2.82 ± 0.75	2.88 ± 0.78	0.395	0.849	0.292
TG/HDL-C	3.06 ± 1.51	2.86 ± 1.55	3.43 ± 1.77	4.01 ± 2.29	<.0001	0.0003	0.012
ApoB/ApoA1	0.87 ± 0.21	0.85 ± 0.19	0.85 ± 0.21	0.88 ± 0.22	0.675	0.870	0.246
hs-CRP[†] (mg/l)	0.97 ± 2.14	0.91 ± 1.99	0.74 ± 1.53	0.87 ± 1.52	0.654	0.358	0.355

Abbreviation: AST, aspartate aminotransferase; SBP, systolic blood pressure; DBP, diastolic blood pressure; TC, total cholesterol; TG, triglyceride; HDL-C, HDL cholesterol; LDL-C, LDL cholesterol; VLDL, very low density lipoprotein; ApoA1, apolipoprotein A1; ApoB, apolipoprotein B; hs-CRP, high sensitivity C-reactive protein. Values are presented as mean±SD. Means with the same letter are not significantly different in row by Bonferroni post hoc test (p>0.05). [†] Log transformed for comparisons to their natural units for presentation
[1)] Analyzed by one-way ANOVA. [2)] Analyzed by Jonckheere-Terpstra trend analysis. [3)] Analyzed by one-way ANOVA, adjusted for alcohol consumption[†]

Table S4. Cardiovascular risk factors along the ALT quartiles

	Quartiles				*p-value* [1)]	*p for trend* [2)]	*Adj. p-value* [3)]
	First (Q1)	Second (Q2)	Third (Q3)	Fourth (Q4)			
SBP (mmHg)	121.31 ± 14.04	123.25 ± 13.56	122.20 ± 11.79	125.74 ± 13.58	0.077	0.038	0.004
DBP (mmHg)	78.68 ± 10.58	79.58 ± 10.02	80.03 ± 9.47	82.31 ± 10.76	0.056	0.011	0.002
TC (mg/dl)	216.99 ± 24.17	216.95 ± 22.01	217.50 ± 26.23	213.59 ± 28.95	0.663	0.159	0.162
TG (mg/dl)	134.74 ± 58.34[c]	148.29 ± 58.25[bc]	158.83 ± 60.11[b]	186.71 ± 74.36[a]	<.0001	<.0001	<.0001
HDL-C (mg/dl)	53.27 ± 12.40[a]	52.07 ± 11.10[a]	51.21 ± 12.25[ab]	46.91 ± 9.79[b]	0.0003	<.0001	0.001
LDL-C (mg/dl)	139.44 ± 21.88	138.32 ± 24.13	139.45 ± 24.74	136.48 ± 25.81	0.778	0.255	0.164
Non HDL-C (mg/dl)	163.72 ± 21.50	164.89 ± 21.73	166.29 ± 27.19	166.68 ± 26.72	0.784	0.494	0.881
VLDL (mg/dl)	33.32 ± 8.97	31.42 ± 5.86	33.17 ± 9.43	36.83 ± 9.31	0.047	0.003	0.027
ApoA1 (g/l)	1.47 ± 0.22	1.47 ± 0.23	1.45 ± 0.27	1.41 ± 0.25	0.266	0.043	0.003
ApoB (g/l)	1.18 ± 0.19	1.21 ± 0.17	1.24 ± 0.22	1.24 ± 0.21	0.099	0.052	0.668
TC/HDL-C	4.23 ± 0.78[b]	4.32 ± 0.85[b]	4.44 ± 1.00[ab]	4.69 ± 0.91[a]	0.001	0.0002	0.025
LDL-C/HDL-C	2.73 ± 0.65	2.77 ± 0.72	2.86 ± 0.79	3.01 ± 0.74	0.024	0.007	0.013
TG/HDL-C	2.79 ± 1.64[b]	3.05 ± 1.52[b]	3.36 ± 1.65[b]	4.22 ± 2.13[a]	<.0001	<.0001	0.001
ApoB/ApoA1	0.82 ± 0.18	0.85 ± 0.20	0.88 ± 0.24	0.90 ± 0.21	0.026	0.007	0.021
hs-CRP[†] (mg/l)	1.18 ± 2.52[b]	0.61 ± 1.28[ab]	0.73 ± 1.71[ab]	0.94 ± 1.40[a]	0.020	0.109	0.245

Abbreviation: ALT, alanine aminotransferase; SBP, systolic blood pressure; DBP, diastolic blood pressure; TC, total cholesterol; TG, triglyceride; HDL-C, HDL cholesterol; LDL-C, LDL cholesterol; VLDL, very low density lipoprotein; ApoA1, apolipoprotein A1; ApoB, apolipoprotein B; hs-CRP, high sensitivity C-reactive protein. Values are presented as mean±SD. Means with the same letter are not significantly different in row by Bonferroni post hoc test (p>0.05). [†] Log transformed for comparisons to their natural units for presentation
[1)] Analyzed by one-way ANOVA. [2)] Analyzed by Jonckheere-Terpstra trend analysis. [3)] Analyzed by one-way ANOVA, adjusted for alcohol consumption[†]

Table S5. Cardiovascular risk factors along the GGT quartiles in male

	Quartiles				*p-value* 1)	*p for trend* 2)	*Adj. p-value* 3)
	First (Q1)	Second (Q2)	Third (Q3)	Fourth (Q4)			
SBP (mmHg)	121.56 ± 12.93[b]	126.08 ± 9.31[ab]	125.70 ± 13.06[ab]	131.12 ± 10.23[a]	0.002	0.0002	0.027
DBP (mmHg)	80.14 ± 9.39[b]	83.57 ± 8.36[ab]	83.40 ± 9.73[ab]	88.63 ± 9.84[a]	0.0004	<.0001	0.008
TC (mg/dl)	206.38 ± 21.83	213.78 ± 19.79	208.07 ± 22.57	219.16 ± 26.82	0.038	0.066	0.069
TG (mg/dl)	144.20 ± 49.90[b]	164.43 ± 62.60[b]	177.95 ± 60.24[ab]	208.91 ± 77.03[a]	<.0001	<.0001	0.002
HDL-C (mg/dl)	46.82 ± 9.00	47.30 ± 9.82	45.09 ± 9.69	48.84 ± 10.42	0.358	0.630	0.086
LDL-C (mg/dl)	131.72 ± 21.11	133.86 ± 23.15	134.09 ± 22.76	137.30 ± 21.96	0.689	0.309	0.508
Non HDL-C (mg/dl)	159.56 ± 18.17	166.49 ± 20.34	162.98 ± 21.10	170.33 ± 25.02	0.094	0.060	0.093
VLDL (mg/dl)	32.71 ± 6.71	33.73 ± 10.19	39.95 ± 10.47	41.03 ± 11.09	0.071	0.008	0.108
ApoA1 (g/l)	1.33 ± 0.19[b]	1.35 ± 0.22[ab]	1.36 ± 0.22[ab]	1.49 ± 0.29[a]	0.012	0.022	0.0003
ApoB (g/l)	1.14 ± 0.14[b]	1.23 ± 0.18[ab]	1.20 ± 0.16[ab]	1.25 ± 0.20[a]	0.014	0.009	0.248
TC/HDL-C	4.51 ± 0.65	4.68 ± 0.92	4.78 ± 0.93	4.65 ± 0.95	0.504	0.610	0.082
LDL-C/HDL-C	2.88 ± 0.56	2.93 ± 0.71	3.09 ± 0.73	2.94 ± 0.79	0.517	0.511	0.109
TG/HDL-C	3.27 ± 1.52[b]	3.73 ± 1.87[ab]	4.19 ± 1.89[ab]	4.55 ± 2.19[a]	0.009	0.0004	0.169
ApoB/ApoA1	0.87 ± 0.16	0.94 ± 0.24	0.91 ± 0.20	0.88 ± 0.24	0.395	0.904	0.042
hs-CRP† (mg/l)	0.40 ± 0.61c	0.39 ± 0.50[bc]	1.45 ± 2.22[ab]	1.06 ± 1.62[a]	0.006	<.0001	0.001

Abbreviation: ALT, alanine aminotransferase; SBP, systolic blood pressure; DBP, diastolic blood pressure; TC, total cholesterol; TG, triglyceride; HDL-C, HDL cholesterol; LDL-C, LDL cholesterol; VLDL, very low density lipoprotein; ApoA1, apolipoprotein A1; ApoB, apolipoprotein B; hs-CRP, high sensitivity C-reactive protein. Values are presented as mean±SD. Means with the same letter are not significantly different in row by Bonferroni post hoc test ($p>0.05$). † Log transformed for comparisons to their natural units for presentation
1) Analyzed by one-way ANOVA. 2) Analyzed by Jonckheere-Terpstra trend analysis. 3) Analyzed by one-way ANOVA, adjusted for alcohol consumption†

Table S6. Cardiovascular risk factors along the GGT quartiles in female

	Quartiles				*p-value* [1)]	*p for trend* [2)]	*Adj. p-value* [3)]
	First (Q1)	Second (Q2)	Third (Q3)	Fourth (Q4)			
SBP (mmHg)	116.24 ± 13.73[b]	122.04 ± 14.22[ab]	120.91 ± 12.72[ab]	125.69 ± 13.16[a]	0.001	0.001	0.079
DBP (mmHg)	74.33 ± 10.44[b]	79.51 ± 10.34[a]	78.08 ± 8.77[ab]	78.67 ± 8.88[ab]	0.009	0.025	0.063
TC (mg/dl)	217.59 ± 29.61	216.81 ± 20.82	219.06 ± 25.97	224.69 ± 27.99	0.294	0.106	0.508
TG (mg/dl)	124.26 ± 57.61[b]	142.77 ± 50.76[ab]	152.34 ± 72.64[ab]	163.78 ± 66.12[a]	0.002	0.000	0.653
HDL-C (mg/dl)	57.26 ± 12.56[a]	52.88 ± 12.57[ab]	53.91 ± 13.29[ab]	50.17 ± 8.81b	0.008	0.001	0.012
LDL-C (mg/dl)	138.73 ± 28.01	139.63 ± 20.35	139.55 ± 23.85	147.48 ± 26.39	0.148	0.064	0.232
Non HDL-C (mg/dl)	160.33 ± 27.17[b]	163.92 ± 22.49[ab]	165.15 ± 25.19[ab]	174.52 ± 27.21[a]	0.012	0.003	0.365
VLDL (mg/dl)	30.99 ± 4.04	32.32 ± 10.07	30.65 ± 4.50	33.33 ± 6.65	0.609	0.452	0.938
ApoA1 (g/l)	1.50 ± 0.22	1.48 ± 0.25	1.54 ± 0.25	1.46 ± 0.19	0.307	0.428	0.041
ApoB (g/l)	1.17 ± 0.21[b]	1.21 ± 0.21[ab]	1.22 ± 0.21[ab]	1.30 ± 0.21[a]	0.009	0.002	0.087
TC/HDL-C	3.93 ± 0.80[b]	4.29 ± 0.92[ab]	4.22 ± 0.84[ab]	4.58 ± 0.87[a]	<.0001	<.0001	0.030
LDL-C/HDL-C	2.52 ± 0.68[b]	2.78 ± 0.72[ab]	2.72 ± 0.73[ab]	3.02 ± 0.76[a]	0.001	0.0002	0.008
TG/HDL-C	2.38 ± 1.46[b]	2.95 ± 1.47[ab]	3.07 ± 1.83[ab]	3.43 ± 1.74[a]	0.002	<.0001	0.505
ApoB/ApoA1	0.79 ± 0.19[b]	0.85 ± 0.22[ab]	0.81 ± 0.20[ab]	0.90 ± 0.20[a]	0.018	0.008	0.020
hs-CRP[†] (mg/l)	1.01 ± 2.69	0.68 ± 1.70	1.13 ± 2.23	0.90 ± 1.20	0.700	0.001	0.075

Abbreviation: ALT, alanine aminotransferase; SBP, systolic blood pressure; DBP, diastolic blood pressure; TC, total cholesterol; TG, triglyceride; HDL-C, HDL cholesterol; LDL-C, LDL cholesterol; VLDL, very low density lipoprotein; ApoA1, apolipoprotein A1; ApoB, apolipoprotein B; hs-CRP, high sensitivity C-reactive protein. Values are presented as mean±SD. Means with the same letter are not significantly different in row by Bonferroni post hoc test (p>0.05). [†] Log transformed for comparisons to their natural units for presentation
[1)] Analyzed by one-way ANOVA. [2)] Analyzed by Jonckheere-Terpstra trend analysis. [3)] Analyzed by one-way ANOVA, adjusted for alcohol consumption[†]
